# 

**Published:** 1889-05

**Authors:** 


### THE AMERICAN JOURNAL —OF— DENTAL SCIENCE,

—EDITED BY—

F. J. S. GORGAS, M. D., D. D. S.

VOLUME XXIII—THIRD SERIES.

SURGEON GEN'L'S OFFICE
136213
LIBRARY

BALTIMORE:
SNOWDEN & COWMAN,
LONDON:
TRUBNER & CO. 60 PATERNOSTER ROW.
1890.

### Index to Volume XXIII.—Third Series.

Antiseptics, - - - - - - - 24
Application and Uses of the Rubber Dam, - - 34
An Entertaining Periodical, - - - - 95
A Surgeon's Notes on Certain Diseases and Malformations of the Teeth, - - - - - 104
A study of Arsenious Acid, - - - - 132
A Victim of Cocaine, - - - - - 139
An Explanation, - - - - - 175
Anæsthetics in Dental Practice, - - - 187
A Few Facts about Platina, - - - - 225
A Case of Rhinolith and two Cases of a Tooth in the Nose, 241
A Real Elixir of Youth, - - - - 284
Axioms in Prosthetic Dentistry, - - - 287
A Tragic Event Recalled, - - - - 329
A Suggestive Case, - - - - - 332
An Improved Way of Using the Hypodermic Method, 334
A Study of the Chemical Composition of the Dental Pulp, 350
An Address on Anæsthetics, - - - 362
Antisepsis in General Practice, - - - - - 414
Aluminum as a Base for Artificial Dentures, - 450
Address Delivered before the Maryland State Dental Association, February 28th, 1889, - - 467
American Academy of Dental Science, - - - 470
A Roland for an Oliver - - - - - 487
A Bill to Regulate the Practice of Medicine in Maryland, 512
A Call to Medical Schools, - - - - • 523
A Valuable Remedy, - - - - - 524
Arsenic, - - - - - - 528
A Simple Regulating Appliance, - - - 574
A Popular Belief in the Contagiousness of Phthisis, 576
Beer Compared with other Alcoholics, - - - 336
Baltimore College of Dental Surgery, - - 568

Bibliographical.

A Text Book of Operative Dentistry, - - 44
Artificial Crown and Bridge work, - - - 282

A Handbook of Dental Pathology for Students and Practitioners, - - - - - 283
Artificial Crown and Bridge-work, - - 478
Artificial Anæsthesia, - - - - - 479
Dental Science, - - - - - - 43
Dental Medicine, - - - - - 142
Dental Anatomy—Human and Comparative, - 478
Goodness of Seeds, - - - - - 575
Practical Electricity in Medicine and Surgery, - 480
Transactions of the American and Southern Dental Associations, - - - - - - 45
Transactions of New York Odontological Society, 45
The Principles and Practice of Dentistry, - - 143
Transactions of the American Dental Association, 479
Vicks' Floral Guide, 1890, - - - - - 527
Chicago Dental Society, - - - - - 42
Crown and Bridge-work, - - - - - 67
Comments on the New Edition (12th) of Harris' Principles and Practice of Dentistry, - - - 87
Charles Wilson Peale as an Inventor of Porcelain Teeth, 91
Care of Children, - - - - - - 145
Cold Water in Facial Neuralgia, - - - 189
Campho Phenique, - - - - - - 229
Cocaine Hallucinations, - - - - - 288
Congenital Syphilis in Infants, - - - 431
Chicago College of Dental Surgery, - - 519
Case of Persistent Facial Neuralgia of Reflex Dental Origin due to an Unerupted Cuspid Tooth, - 356
Chloralamid, - - - - - - - 549
Care of the Deciduous Teeth, - - - - 554
Cases of Epulis, - - - - - - - 563
Dental Law of Colorado, - - - - - 40
Department of the Interior, Census Office, - - 94
Dental Decay, - - - - - - - 97
Dental Irregularities of the Native Races, - - 100
Dissemination of the Knowledge of Dental Hygiene Among the Masses, - - - - - 163
Dentition as Affected by Rickets, - - - 276
Death after taking Nitrous Oxide, - - - 288

Death from Chloroform, - - - - - 384
Dental Education, - - - - - - 456
Dental Caries, - - - - - 461
Dislocation Inferior Maxillary, - - - - - 524
Etiology of Caries, - - - - - - 154
Escharotics and Coagulants, - - - - 217
Excessive Hemorrhage after Extraction of Teeth—A Case, 464
Ether, Chloroform or Nitrous Oxide, - - - 529
Eruption of Teeth in Old Age, - - - 572
Filling Teeth With Vulcanite, - - - - 144
Faults of Copper Amalgam, - - - 190
Filling Proximal Cavities in Bicuspids and Molars, - 201
Foreign Dentists in England, - - - 278
Fatal Case of Nitrous Oxide Anæsthesia, - - 312
Filling Root Canals of Human Teeth, - - 314
Failures, - - - - - - 359
Facial Neuralgia and Allied Neurosis, - - 517
Georgia State Dental Society, - - - - 337
Gonorrhœa at Five Years of Age, - - - 383
Georgia State Dental Society, - - - - 398
How are Deciduous Teeth Cast off, - - 321
Impression Taking - - - - - - 48
Influence of Certain Medical Agents on the Bacillus of Tubercle in Man, - - - - - 96
Infection by Physicians, - - - - 565
Looking Forward, - - - - - 433
Methyl Chloride for Local Anæsthesia, - - 38
Making every Child a Chemist, - - - 278
Make the Foundations Firm, - - - 325
Missouri State Dental Association, - - - 520
Neurectomy of the Inferior Branch of the Fifth Nerve, 65
National Association of Dental Examiners, - - 193
National Association of Dental Faculties, - - 197
Neurectomy for Facial Neuralgia, with Recovery, - 247
Nitrous Oxide Narcosis and the Blood, - - 286
Neglected Advantages, - - - - - 303
Notes of a Case of Sclerotitis Apparently of Dental Origin, 309
Non Metallic Plastic Materials for filling Teeth, - 481
On Plastic Fillings, - - - - - 31

One Way of Increasing a Dentists' Usefulness, - 368
OBITUARY.
Dr. Charles F. Dinger, - - - - 43
Dr. Philippe Ricord. - - - - 281
Elisha Parsons, M. D., D. D. S., - - 330
F. H. Rehwinkel, M. D., D. D. S., - - - 140
Progress of Dentistry During Year Ending May 1st, 1889, 117
Persistent Headaches, and How to Cure Them, - 385
Pyorrhœa Alveolaris, - - - - 444
Porcelain Filling, - - - - - 516
Professional Ethics and Honor, - - - 540
Restoring Discolored Teeth, - - - - 112
Reflex Neurosis in Relation to Dental Pathology, - 289
Short Notes on Diseases of the Palate, - - 20
Sensitive Dentine, - - - - - 78
Saccharine, - - - - - - 138
Separate the Operative from the Mechanical, - 170
Some Incidents of Practice in Mexico, - - - 231
Some Notes on the Early Art of Extracting Teeth, 249
Syphilitic Teeth, - - - - - 285
Source of Colors, - - - - - 432
Some Further Notes on the Invention of Porcelain Teeth by the Late Chas. W. Peale, - - - 473
Still Another New Hypnotic, - - - 558
Synthetic Carbolic Acid, - - - - 572
The Odontological Society of Great Britain, - - 1
The Dentists of the Day - - - - 14
The Nervous Patient, - - - - 27
Teeth of Some Animals, - - - - 46
The New Epoch in Medicine, - - - 49
The National Association of Dental Examiners, - 86
The Johnstown Calamity, - - - - 90
The Southern Dental Association, - - - 181
The Colorado Dental Law, - - - - 182
The Texas Dental Law, - - - - 184
The Micro-Organisms of the Mouth and their Association with Disease, - - - - - 212
The Dental Protective Association of the United States, 232
The University of Maryland Dental Department, - 238

The Dental Meetings at Saratoga, - - - 239
The Preparation and Filling of Roots at one Sitting, 263
The Importance of a Thorough Mechanical Training, 299
The History of the University of Maryland Dental Department. - - - - - 327
The Mortality Among Nurses - - - 335
The Cocoanut as a Tæniacide, - - - - 336
To Whom it may Concern, - - - 372
The Dedication of Meharry Dental and Pharmaceutical College, - - - - - 378
The Mouth in Backward Children (Imbecile) of the Mongolian Type, - - - - - 379
The Hour at which Death most Usually Occurs, - 427
The Amendments to the Maryland State Dental Law, 477
Tenth International Medical Congress, Berlin, 1890, - 496
Tenth International Medical Congress, Berlin, - 521
Twentieth Annual Meeting of the South Carolina State Dental Association, - - - - 521
The Teeth and Orad Cavity of Pregnant Women, - 559
Tobacco-Smoking and Disease, - - - 566
The Way to Educate Medical Students, - - 570
The Stomach-brush, - - - - 573
The Influence of Hereditary in the Causation of Tuberculosis of Bone, - - - - 575
Thymol Dentific, - - - - - 576
University of Maryland Dental Department, - 91
" " " " - - 138
" " " " - - 475
Washing Amalgam, - - - - - 45
White Chlora-Percha, - - - - 528
Well Formed Children, - - - - 525

### TO READERS AND CORRESPONDENTS.

Communications intended for publication, and exchange journals and papers, should be addressed to the Editor of THE AMERICAN JOURNAL OF DENTAL SCIENCE, No. 845 N. Eutaw St. Baltimore, Md. All letters relating to the business of the JOURNAL, or containing cash remittances, to be directed to Messrs. Snowden & Cowman, No. 9 W. Fayette Street, Baltimore, Md. Articles for publication should be received by the Editor at least two weeks previous to the first of the month on which the number in which it is designed for them to appear, is issued.

### CONTENTS:

Chicago Dental Society. . . . . . . . . . . 42

OBITUARY.

Dr. Charles F. Dinger. . . . . . . . . . 43

BIBLIOGRAPHICAL.

Dental Science. . . . . . . . . . . 43
A Text Book of Operative Dentistry. . . . . . . 44
Transactions of the American and Southern Dental Associations. 45
Transactions of New York Odontological Society. . . . 45

MONTHLY SUMMARY.

Washing Amalgam. . . . . . . . . . . . 45

Teeth of Some Animals. . . . . . . . . . . 46

Impression Taking. . . . . . . . . . . . 48

☞ THE AMERICAN JOURNAL OF DENTAL SCIENCE will contain fifty pages of reading matter in each number, making a volume of about Six Hundred pages,

EXCLUSIVE OF ADVERTISEMENTS.

